# Differential Response of Human Dendritic Cells upon Stimulation with Encapsulated or Non-Encapsulated Isogenic Strains of *Porphyromonas gingivalis*

**DOI:** 10.3390/ijms25084510

**Published:** 2024-04-20

**Authors:** Samanta Melgar-Rodríguez, Alan Polanco, Jearitza Ríos-Muñoz, Michelle García, Alfredo Sierra-Cristancho, Luis González-Osuna, Jaime Díaz-Zúñiga, Paola Carvajal, Rolando Vernal, Denisse Bravo

**Affiliations:** 1 Periodontal Biology Laboratory, Faculty of Dentistry, Universidad de Chile, Santiago 8380492, Chile; smelgar@odontologia.uchile.cl (S.M.-R.); alan.polanco.95@gmail.com (A.P.); jrios@odontologia.uchile.cl (J.R.-M.); michelitapao@gmail.com (M.G.); asierra@odontologia.uchile.cl (A.S.-C.); luisgodont@gmail.com (L.G.-O.); jdiaz@odontologia.uchile.cl (J.D.-Z.); pcarvajal@odontologia.uchile.cl (P.C.); 2 Department of Conservative Dentistry, Faculty of Dentistry, Universidad de Chile, Santiago 8380492, Chile; 3 Biomedical Research Center, Faculty of Medicine, Universidad de Valparaíso, Valparaíso 2360102, Chile; 4 Faculty of Dentistry, Universidad Andrés Bello, Santiago 8370035, Chile; 5 Laboratorio de Interacciones Microbianas, Faculty of Dentistry, Universidad Andrés Bello, Santiago 8370035, Chile

**Keywords:** *Porphyromonas gingivalis*, capsule, dendritic cells, cytokines, transcription factors

## Abstract

During periodontitis, the extracellular capsule of *Porphyromonas gingivalis* favors alveolar bone loss by inducing Th1 and Th17 patterns of lymphocyte response in the infected periodontium. Dendritic cells recognize bacterial antigens and present them to T lymphocytes, defining their activation and polarization. Thus, dendritic cells could be involved in the Th1 and Th17 response induced against the *P. gingivalis* capsule. Herein, monocyte-derived dendritic cells were obtained from healthy individuals and then stimulated with different encapsulated strains of *P. gingivalis* or two non-encapsulated isogenic mutants. Dendritic cell differentiation and maturation were analyzed by flow cytometry. The mRNA expression levels for distinct Th1-, Th17-, or T-regulatory-related cytokines and transcription factors, as well as TLR2 and TLR4, were assessed by qPCR. In addition, the production of IL-1β, IL-6, IL-23, and TNF-α was analyzed by ELISA. The encapsulated strains and non-encapsulated mutants of *P. gingivalis* induced dendritic cell maturation to a similar extent; however, the pattern of dendritic cell response was different. In particular, the encapsulated strains of *P. gingivalis* induced higher expression of IRF4 and NOTCH2 and production of IL-1β, IL-6, IL-23, and TNF-α compared with the non-encapsulated mutants, and thus, they showed an increased capacity to trigger Th1 and Th17-type responses in human dendritic cells.

## 1. Introduction

Periodontitis is an inflammatory disease elicited by periodontopathic bacteria attached to the tooth that colonize the subgingival environment [1]. Periodontitis is considered a public health problem given that it is the leading cause of tooth loss, its severe forms affect between 5.8% and 49.7% of the global adult population, with increasing incidence, and it compromises the quality of life of individuals [2,3,4,5]. In addition, the high prevalence of severe forms of periodontitis contributes to the global burden of other chronic non-communicable diseases, such as cardiovascular disease, diabetes mellitus, Alzheimer’s disease, and adverse pregnancy outcomes [6,7,8].

The pathogenicity of the bacterial members of the subgingival dysbiotic biofilm relies mainly on the activation of host immuno-inflammatory mechanisms in the infected periodontium [9,10]. These bacterial-host interactions at the biofilm-periodontium interface trigger changes in the infiltrating cell immune composition, where the predominance of pro-inflammatory Th1 and Th17 lymphocytes and the decreased number of anti-inflammatory Th2 and T regulatory lymphocytes are associated with the pathogenesis of periodontitis [11,12,13]. Consequently, the increment in Th1-type cytokines, such as IL-1β, IL-12, IFN-γ, and TNF-α, and Th17 cytokines, such as IL-6 and IL-23, favors the accumulation of osteolytic factors that induce osteoclastogenesis and lead to the destruction of the tooth-supporting alveolar bone [11,14,15].

Only a limited number have been associated with periodontitis from more than 700 bacterial species identified in the subgingival biofilm [16,17]. Among them, *Porphyromonas gingivalis* expresses several virulence factors that may contribute to periodontitis pathogenesis, including fimbriae, lipopolysaccharide, gingipains, enzymes, outer membrane proteins, and extracellular capsule [18,19,20,21]. Remarkably, the extracellular capsule is critical in the virulence of *P. gingivalis* [22,23]. As such, encapsulation of *P. gingivalis* contributes to defining the dysbiotic changes that occur during periodontitis and determining the pattern of immune response deployed in infected tissues [24,25,26,27]. During periodontitis, encapsulation of *P. gingivalis* promotes osteoclast differentiation and alveolar bone loss by inducing Th1 and Th17 immunity patterns [26,28,29]. Periodontal inoculation of encapsulated strains of *P. gingivalis* in mice leads to marked T-cell infiltration and favors their differentiation towards the Th1 and Th17 subsets, with pro-inflammatory and osteolytic activities [11,26,30]. Emphasizing this, when experimental periodontitis was induced using a capsular-defective knockout mutant of *P. gingivalis*, less osteoclastogenesis and alveolar bone resorption were observed, compared with the encapsulated isogenic strain [26]. Notably, this decreased tooth-supporting alveolar bone loss was attributed to less differentiation and activation of Th17 lymphocytes in periodontal lesions [26].

Recently, it has been proposed that pathological immune commitment begins with the first cells contacting and recognizing antigens [31,32,33]. Indeed, T-cell polarization could even be defined before antigen presentation when the microbial antigens induce a particular dendritic cell subset [31,33,34,35]. Certainly, dendritic cells could selectively acquire particular functional characteristics by recognizing capsular antigens of *P. gingivalis* and consequently, could influence the polarization and function of T lymphocytes and the nature of periodontal immunity [28,29]. In this context, distinct subsets of dendritic cells are recognized based on the transcription factors and the pattern of cytokines they express [36,37,38]. The conventional type 1 (cDC1) subset expresses the transcription factors basic leucine zipper ATF-like transcription factor 3 (BATF3) and interferon regulatory factor 8 (IRF8), the conventional type 2 (cDC2) subset expresses interferon regulatory factor 4 (IRF4), and the plasmacytoid (pDC) subset expresses IRF8 [35,38,39,40,41,42,43,44]. All these dendritic cell subsets produce IL-1β, IL-12, IFN-γ, and TNF-α, polarizing T cells towards the Th1 phenotype; nevertheless, the overexpression of the transcription factors IRF4 and neurogenic locus notch homolog protein 2 (NOTCH2) in the cDC2s leads them to also produce IL-6 and IL-23, priming Th17 lymphocyte differentiation [35,39,43,44,45].

This study aimed to elucidate whether dendritic cells change the expression pattern of the transcription factors that define their functional polarization and the cytokine profile secreted when exposed to the extracellular capsule of *P. gingivalis*. Thus, we analyzed the expression of the transcription factors BATF3, IRF4, IRF8, and NOTCH2 and the cytokines IL-1β, IL-6, IL-10, IL-12, IL-23, IFN-γ, TGF-β1, and TNF-α in dendritic cells induced with the encapsulated W50 strain of *P. gingivalis* or its non-encapsulated isogenic mutants GPA and GPC. We hypothesized that dendritic cells stimulated with the encapsulated W50 strain of *P. gingivalis* upregulate the production of the Th17-related cytokines IL-6 and IL-23, and transcription factors IRF4 and NOTCH2, compared with the same cells stimulated with the GPA and GPC mutants defective in the extracellular capsule.

## 2. Results

### 2.1. Flow Cytometric Analysis of Monocyte Purification and Dendritic Cell Differentiation

**Figure 1:**
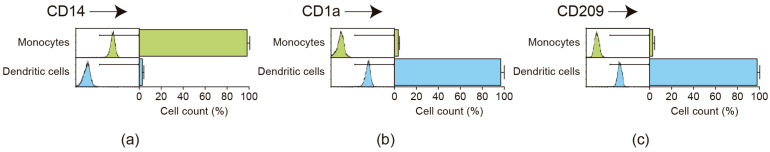
Monocyte purification and dendritic cell differentiation. Flow cytometry analysis of the expression of (**a**) CD14, demonstrating the purity of monocytes isolated from peripheral blood (CD14-positive cells); (**b**) CD1a; and (**c**) CD209 (DC-SIGN), demonstrating the efficiency of differentiation of monocytes towards dendritic cells (CD1a- and CD209-positive cells) in the presence of rhGM-CSF and rhIL-4. The data from each experiment were expressed as the percentage of positive cells over the total and shown as mean ± SD from 8 independent experiments. Each experiment was performed in duplicate.

The present study used human blood monocyte-derived dendritic cells to analyze differential responses upon *P. gingivalis* infection. The isolation of peripheral blood monocytes and their differentiation toward untouched dendritic cells was demonstrated by staining the CD14, CD1a, and CD209 (DC-SIGN) surface markers and analyzing their expression by flow cytometry (Figure 1). A highly purified (98.3 ± 2.4%) population of CD14^+^ monocytes was obtained. These monocytes differentiated at a high frequency into dendritic cells upon culture in the presence of rhGM-CSF and rhIL-4, as demonstrated by the appearance of the CD1a (96.6 ± 3.1%) and CD209 (97.8 ± 2.4%) antigens and the concomitant loss of the monocyte marker CD14. Dendritic cell viability was greater than 97.9 ± 1.6%, as determined by trypan blue exclusion.

### 2.2. Flow Cytometric Analysis of Dendritic Cell Maturation

**Figure 2:**
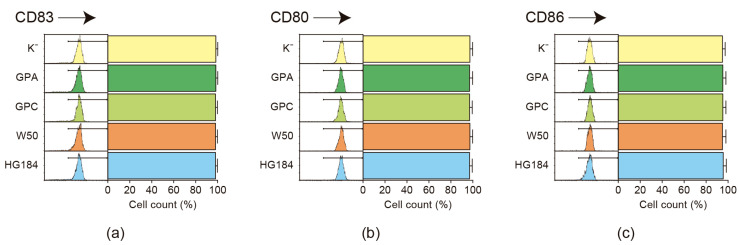
Dendritic cell maturation. Flow cytometry analysis of the expression of (**a**) CD83, a marker of dendritic cell maturation; (**b**) CD80; and (**c**) CD86, costimulatory signals necessary for T-cell activation during antigen presentation, demonstrating the dendritic cell maturation after a 2-day stimulation with the different strains of *P. gingivalis* (MOI = 100). The data from each experiment were expressed as the percentage of positive cells over the total and shown as mean ± SD from 8 independent experiments. Each experiment was performed in duplicate. K^−^: non-encapsulated ATCC^®^33277™ wild-type strain; GPA: non-encapsulated ΔPG0116-PG0120 mutant strain; GPC: non-encapsulated ΔPG0109-PG0118 mutant strain; W50: encapsulated ATCC^®^53978™ wild-type strain; HG184: encapsulated HG184 wild-type strain.

To compare the dendritic cell maturation levels after stimulation with the different strains of *P. gingivalis*, the CD83, CD80, and CD86 cell surface expression levels were analyzed by flow cytometry (Figure 2). No differences were detected in the maturation levels of dendritic cells between the different bacterial strains, as demonstrated by the increased expression of CD83 (>94.6%), CD80 (>97.8%), and CD86 (>96.8%) antigens, indicating that the encapsulated and non-encapsulated strains of *P. gingivalis* induce dendritic cell maturation to a similar extent.

### 2.3. Toll-like Receptor Expression in P. gingivalis-Infected Dendritic Cells

**Figure 3:**
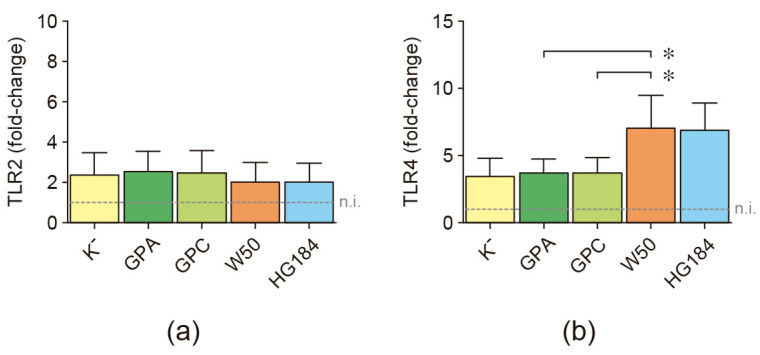
TLR2 and TLR4 expression. (**a**) TLR2 and (**b**) TLR4 mRNA expression quantified by qPCR in dendritic cells stimulated with the different strains of *P. gingivalis* (MOI = 100). For relative expression, the TLR mRNA expression in non-infected dendritic cells was considered 1 as a reference for fold-change in expression (n.i.). Data are represented as mRNA fold-change and shown as mean ± SD from 8 independent experiments. Each experiment was performed in duplicate. TLR: Toll-like receptor; K^−^: non-encapsulated ATCC^®^33277™ wild-type strain; GPA: non-encapsulated ΔPG0116-PG0120 mutant strain; GPC: non-encapsulated ΔPG0109-PG0118 mutant strain; W50: encapsulated ATCC^®^53978™ wild-type strain; HG184: encapsulated HG184 wild-type strain. * *p* < 0.05 for the W50 strain compared to the GPA and GPC mutants of *P. gingivalis*.

To demonstrate that dendritic cells are differentially activated upon stimulation with the different strains of *P. gingivalis* despite their maturation levels being similar, the expression levels of the surface receptors TLR2 and TLR4, involved in the recognition of *P. gingivalis* antigens, were quantified (Figure 3). When the encapsulated W50 wild-type strain of *P. gingivalis* was used for dendritic cell stimulation, significantly higher expression levels of TLR4 were detected, compared to dendritic cells stimulated with the non-encapsulated GPA or GPC isogenic mutant strains. These increased levels of TLR4 expression upon W50 stimulation were similar to those detected in dendritic cells stimulated with the HG184 strain of *P. gingivalis*, used as an encapsulated control. Similar expression of TLR4 was also found in dendritic cells stimulated with GPA or GPC mutants and cells stimulated with the ATCC^®^33277™ (K^−^) strain of *P. gingivalis*, used as a non-encapsulated control. No differences were detected in the mRNA expression for TLR2.

### 2.4. Transcription Factor Expression in P. gingivalis-Infected Dendritic Cells

**Figure 4:**
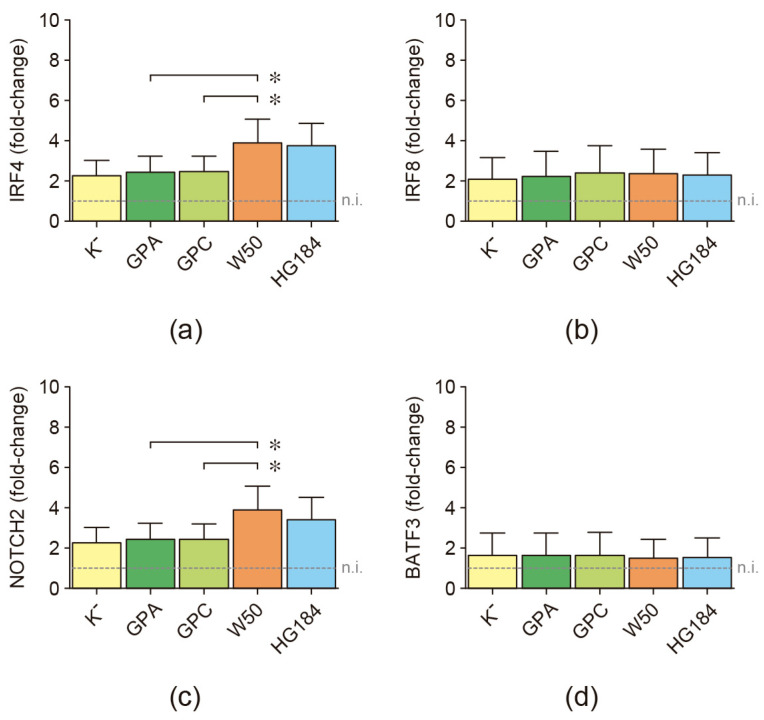
Transcription factor expression. (**a**) IRF4, (**b**) IRF8, (**c**) NOTCH2, and (**d**) BATF3 mRNA expression quantified by qPCR in dendritic cells stimulated with the different strains of *P. gingivalis* (MOI = 100). For relative expression, the transcription factor mRNA expression in non-infected dendritic cells was considered 1, as a reference for fold-change in expression (n.i.). Data are represented as mRNA fold-change and shown as mean ± SD from 8 independent experiments. Each experiment was performed in duplicate. IRF4: interferon regulatory factor 4; IRF8: interferon regulatory factor 8; NOTCH2: neurogenic locus notch homolog protein 2; BATF3: basic leucine zipper ATF-like transcription factor 3. K^−^: non-encapsulated ATCC^®^33277™ wild-type strain; GPA: non-encapsulated ΔPG0116-PG0120 mutant strain; GPC: non-encapsulated ΔPG0109-PG0118 mutant strain; W50: encapsulated ATCC^®^53978™ wild-type strain; HG184: encapsulated HG184 wild-type strain. * *p* < 0.05 for the W50 strain compared to the GPA and GPC mutants of *P. gingivalis*.

As a first approximation in the analysis of a possible association between the encapsulation of *P. gingivalis* and induction of a particular dendritic cell subset, the expression levels for IRF4, IRF8, NOTCH2, and BATF3 mRNAs were quantified after their stimulation with the different strains of *P. gingivalis* (Figure 4). IRF4, IRF8, and NOTCH2 expression were induced in dendritic cells from all the analyzed individuals; however, BATF3 remained undetectable in one individual under any experimental conditions. When the encapsulated W50 wild-type strain of *P. gingivalis* was used for dendritic cell stimulation, significantly higher expression levels of IRF4 and NOTCH2 were detected, compared to dendritic cells stimulated with the GPA or GPC mutant strains, indicative of an association between the encapsulation of *P. gingivalis* and the induction of a Th17-type response in these cells. Indeed, the increased IRF4 and NOTCH2 expression levels detected in the W50-stimulated dendritic cells were similar to those detected in the HG184-stimulated dendritic cells. These transcription factors were also expressed similarly between GPA- or GPC-stimulated and K^−^-stimulated dendritic cells. No differences were detected in the mRNA expression for IRF8 and BATF3.

### 2.5. Cytokine Expression in P. gingivalis-Infected Dendritic Cells

**Figure 5:**
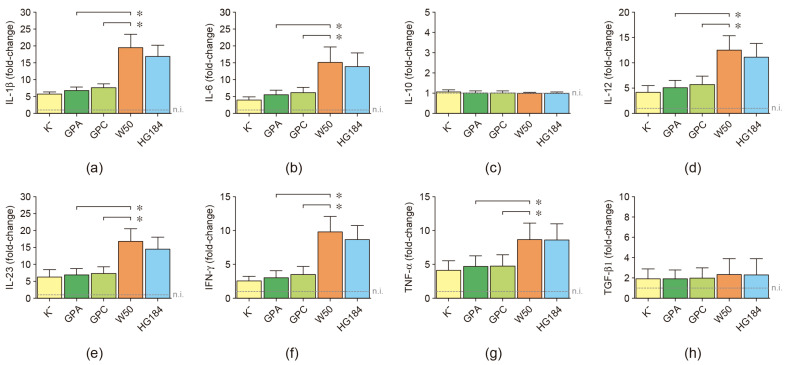
Cytokine expression. (**a**) IL-1β, (**b**) IL-6, (**c**) IL-10, (**d**) IL-12, (**e**) IL-23, (**f**) IFN-γ, (**g**) TNF-α, and (**h**) TGF-β1 mRNA expression quantified by qPCR in dendritic cells stimulated with the different strains of *P. gingivalis* (MOI = 100). For relative expression, the cytokine mRNA expression in non-infected dendritic cells was considered 1, as a reference for fold-change in expression (n.i.). Data are represented as mRNA fold-change and shown as mean ± SD from 8 independent experiments. Each experiment was performed in duplicate. IL: interleukin; IFN: interferon; TNF: tumor necrosis factor; TGF: transforming growth factor; K^−^: non-encapsulated ATCC^®^33277™ wild-type strain; GPA: non-encapsulated ΔPG0116-PG0120 mutant strain; GPC: non-encapsulated ΔPG0109-PG0118 mutant strain; W50: encapsulated ATCC^®^53978™ wild-type strain; HG184: encapsulated HG184 wild-type strain. * *p* < 0.05 for the W50 strain compared to the GPA and GPC mutants of *P. gingivalis*.

To ratify the association between the encapsulation of *P. gingivalis* and induction of a particular dendritic cell subset, the mRNA expression for IL-1β, IL-6, IL-10, IL-12, IL-23, IFN-γ, TGF-β1, and TNF-α was quantified by RT-qPCR (Figure 5). When the encapsulated W50 wild-type strain of *P. gingivalis* was used for dendritic cell stimulation, significantly higher expression levels of IL-1β, IL-12, IFN-γ, and TNF-α (Th1-type cytokines), and IL-6 and IL-23 (Th17-type cytokines) were detected, compared to dendritic cells stimulated with the non-encapsulated GPA or GPC mutant strains. The increased levels of these Th1 and Th17-type cytokines detected in the dendritic cells stimulated with the W50 strain were similar to those detected in dendritic cells stimulated with the encapsulated HG184 strain of *P. gingivalis*. These cytokines were also expressed similarly between GPA- or GPC-stimulated and K^−^-stimulated dendritic cells. No differences were detected in the mRNA expression for IL-10 and TGF-β1.

### 2.6. Cytokine Secretion in P. gingivalis-Infected Dendritic Cells

**Figure 6:**
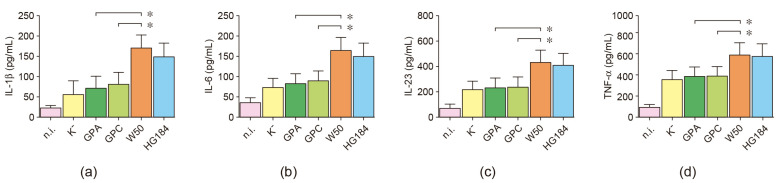
Cytokine secretion. (**a**) IL-1β, (**b**) IL-6, (**c**) IL-23, and (**d**) TNF-α secretion quantified by ELISA in dendritic cells stimulated with the different strains of *P. gingivalis* (MOI = 100). Data are represented as pg/mL and shown as mean ± SD from 6 independent experiments. Each experiment was performed in duplicate. IL: interleukin; TNF: tumor necrosis factor, n.i.: non-infected; K^−^: non-encapsulated ATCC^®^33277™ wild-type strain; GPA: non-encapsulated ΔPG0116-PG0120 mutant strain; GPC: non-encapsulated ΔPG0109-PG0118 mutant strain; W50: encapsulated ATCC^®^53978™ wild-type strain; HG184: encapsulated HG184 wild-type strain. * *p* < 0.05 for the W50 strain compared to the GPA and GPC mutants of *P. gingivalis*.

The association between the encapsulation of *P. gingivalis* and the upregulation of Th1- and Th17-type cytokines in infected dendritic cells was also demonstrated at the protein level (Figure 6). When the encapsulated W50 wild-type strain of *P. gingivalis* was used for dendritic cell stimulation, significantly higher secreted levels of IL-1β, IL-6, IL-23, and TNF-α were detected, compared to dendritic cells stimulated with the non-encapsulated GPA or GPC mutant strains. The increased levels of these Th1- and Th17-type cytokines detected upon W50 stimulation were similar to those detected in dendritic cells stimulated with the encapsulated HG184 strain of *P. gingivalis*. These cytokines were also secreted similarly between GPA- or GPC-stimulated and K^−^-stimulated dendritic cells.

## 3. Discussion

This study analyzed the role of the extracellular capsule of *P. gingivalis* in the immune response deployed by human dendritic cells. In particular, it was analyzed whether the W50 strain of *P. gingivalis*, belonging to the K1 capsular serotype, mainly induces Th1 and Th17 patterns of immune response, characteristic of periodontitis. It was shown that, compared to its non-encapsulated isogenic mutants, the encapsulated W50 strain induces greater production of the cytokines IL-1β, IL-6, IL-12, IL-23, IFN-γ, and TNF-α, as well as greater expression of the transcription factors IRF4 and NOTCH2, distinctive of Th1 and Th17-type immune responses.

These findings are consistent with the higher immunogenicity and pathogenicity previously described for serotype K1 of *P. gingivalis*, compared to the other capsular serotypes [28,29,46,47]. Indeed, in human dendritic cells stimulated with different capsular or non-capsular strains of *P. gingivalis*, a greater expression of cytokines of the Th1 and Th17 profiles was detected in the presence of the K1 serotype [46,47]. Similarly, T lymphocytes stimulated with autologous dendritic cells exposed to *P. gingivalis* serotype K1 expressed higher levels of cytokines and transcription factors related to the Th1 and Th17 lymphocyte subtypes, compared to the same cells stimulated with the other capsular serotypes [28]. The *P. gingivalis* serotype K1 revealed more potent immunogenicity by inducing cytokine production at a lower MOI than the others and, particularly, higher levels of the cytokines IL-1β, IL-6, IL-12, IL-17A, IL-23, IFN-γ, TNF-α, and TNF-β and the transcription factors T-bet and RORC2, directly related to Th1 and Th17 patterns of T-cell differentiation and function [28]. Furthermore, this higher immunogenicity attributed to serotype K1 was directly associated with its increased pathogenic potential when it elicited higher production of the osteolytic factor called receptor activator of nuclear factor κB ligand (RANKL) in primed T lymphocytes, which is a potent inducer of osteoclastogenesis and bone loss [29]. These findings reveal immunogenic differences attributed to the extracellular capsule between the different *P. gingivalis* strains analyzed, which would be related to variations in the composition and structure of their capsular polysaccharides [48,49]. However, these *P. gingivalis* strains not only differ in genes encoding the different proteins involved with capsular conformation [50,51]. In fact, these bacterial strains are characterized by having a totally different genetic background, such that genes related to other virulence factors could also vary between them. In this sense, along with capsular variations, these *P. gingivalis* strains could also vary in other virulence factors that could contribute, at least in part, to the immunogenic differences detected in dendritic cells and T lymphocytes.

*P. gingivalis* is one of the prime etiological agents related to periodontitis onset and progression [52]. In fact, *P. gingivalis* is found in 85.75% of subgingival plaque samples from patients with chronic periodontitis [18]. The high pathogenicity of *P. gingivalis* lies in the diverse virulence factors that it expresses, among which the extracellular capsule stands out. The capsule is involved in the adherence of *P. gingivalis* to oral mucosa and tooth surfaces; thus, increased encapsulation provides resistance to the flow of saliva and gingival crevicular fluid and favors bacterial colonization [53]. Furthermore, the presence and type of capsule have been associated with the initial adhesion of *P. gingivalis* to periodontal pocket epithelial cells and with the coaggregation of *P. gingivalis* to other periodontopathogenic bacteria, such as *Fusobacterium nucleatum* [54,55]. According to the capsule, *P. gingivalis* displays at least six distinct serotypes, comprising K1–K6. Among them, the W50 strain belonging to the K1 serotype has a thicker capsule, and it is the most frequently detected in periodontitis patients [56], which is why it was studied herein. As an encapsulation-positive control, the HG184 strain belonging to the K2 capsular serotype was used because it is one of the original *P. gingivalis* strains in which serotypic variants were described [57,58]. As a negative control, the non-encapsulated ATCC^®^33277™ strain was selected since it is the most frequently isolated from periodontal sites of individuals that harbor *P. gingivalis* [56]. In addition, the ATCC^®^33277™ strain has been mainly associated with less severe cases of periodontitis and periodontal health [59]. Indeed, the ATCC^®^33277™ strain has been proposed to be the healthier *P. gingivalis* strain with reduced virulence characteristics [59,60].

The present study focused on the induction of dendritic cells, comparing the wild-type W50 strain belonging to serotype K1 of *P. gingivalis* with two isogenic mutant strains constructed from it. It is noteworthy that the compared strains correspond to genetic modifications related only to the genetic locus that encodes the extracellular capsule; particularly, strains GPA and GPC are deletions in the ΔPG0116-PG0120 and ΔPG0109-PG0118 regions, respectively [50]. Consequently, the induction of Th1 and Th17-type response patterns reported here in dendritic cells can be attributed exclusively to the extracellular capsule, rather than to some other virulence factor as in previous studies [28,29,46,47]. By extension, the greater pathogenicity of *P. gingivalis* serotype K1 that was reported using an animal model of periodontitis and the same bacterial strains used in the present study [26] can be consistently explained, at least in part, by the Th1 and Th17 patterns generated in the early stages of the immune response, when dendritic cells recognize the capsular antigens. This conclusion requires ratification by analyzing the differential immune response triggered by the capsule of *P. gingivalis* directly in T lymphocytes, given that the findings reported in the present study only allow us to speculate on these differences. We must highlight this limitation of our study, given that we did not analyze the effect of the *P. gingivalis* capsule on T lymphocytes, which, by definition, are the cells directly responsible for developing an immune response with a particular Th pattern. Furthermore, unlike the broader repertoire of cytokines T lymphocytes can produce, we only analyzed a limited number of cytokines among those that dendritic cells produce [61,62]. Even so, we consider that our study is an essential contribution to defining the role of the extracellular capsule of *P. gingivalis* in the induction of a pro-inflammatory and osteolytic immune response since dendritic cells contribute significantly to determining the type of T lymphocyte response deployed after antigen presentation.

In the initial stages of the periodontal immune response, dendritic cells play a fundamental role in recognizing *P. gingivalis* through the TLRs they express [63]. In particular, overexpression of TLR4 has been associated with the recognition of and response to serotypes K1 and K2 of *P. gingivalis* [46]. According to the data presented here, the absence of an extracellular capsule in the isogenic GPA and GPC mutants of *P. gingivalis* is related to lower expression of TLR4 in dendritic cells compared to the wild-type bacterial strain, which is consistent with previous reports. In turn, this would allow linking TLR4-mediated extracellular capsule signaling with the Th1 and Th17 types of responses we observed. After being activated, dendritic cells selectively acquire particular phenotypic and functional characteristics, and the presence of the extracellular capsule in *P. gingivalis*, together with cell signaling via TLR4, could be related to the induction of a particular dendritic cell phenotype [41]. In this sense, the overexpression of IRF4 and NOTCH2 that we observed in dendritic cells pulsed with the wild-type W50 strain compared to its isogenic mutants GPA and GPC could be related to the induction of dendritic cells belonging to the cDC2 subset. Indeed, it has been reported that the cDC2 population that expresses the IRF4 transcription factor produces IL-1β, IL-12, IFN-γ, and TNF-α and, consequently, favors the induction of Th1 lymphocytes during antigen presentation. Conversely, the overexpression of IRF4 and NOTCH2 in cDC2s turns them into producers of IL-6 and IL-23, which was observed in this study and would be associated with a bias towards Th17 lymphocyte differentiation [35,39,43,44,45].

During periodontitis, the progressive destruction of teeth-supporting tissues mainly results from Th1 and Th17 patterns of immune response [11]. In particular, Th1 and Th17 lymphocytes together produce IL-1β, IL-6, IL-12, IL-17A, IL-23, IFN-γ, and TNF-α, which leads to alveolar bone resorption through the induction of RANKL production in fibroblasts and osteoblasts. Furthermore, Th17 cells can also produce RANKL and thus directly induce periodontal alveolar bone loss [11]. According to the data of the present study, dendritic cells induced by *P. gingivalis*, especially by capsular strains belonging to serotype K1, could be related to periodontal bone loss since they would favor TLR4-mediated polarization of IRF4^+^NOTCH2^+^ cDC2s and, during the presentation of antigens derived from *P. gingivalis*, they could participate in the induction of Th1 and Th17 types of response with osteolytic potential. This is a speculation projected from our data, which we have not demonstrated in this study. In our opinion, the value of the present study is that it contributes to elucidating the immune basis that explains, at least partly, the increased pathogenic potential of serotype K1 of *P. gingivalis*. By extension, it shows how certain strains belonging to particular bacterial species contribute to the etiopathogenesis of periodontitis, while others do not. Taken together, the data presented in this study and those previously reported allow us to establish that the pathogenic differential of the different capsular serotypes of *P. gingivalis* is based on the induction of a periodontal immune response with a predominance of Th1 and Th17, which would be deployed from the first immune events when dendritic cells recognize *P. gingivalis* capsular antigens.

## 4. Materials and Methods

### 4.1. Porphyromonas gingivalis Strains

In the present study, the following *P. gingivalis* strains were used: the encapsulated wild-type W50 (ATCC^®^53978™) strain, belonging to the K1 capsular serotype, and two W50-based knockout mutants termed GPA (ΔPG0116-PG0120) and GPC (ΔPG0109-PG0118), defective in extracellular encapsulation [50]. The wild-type HG184 strain, belonging to the K2 capsular serotype, was used as an encapsulated control. The wild-type ATCC^®^33277™ strain was used as a non-encapsulated control. The bacterial strains were cultured on 5% horse blood agar (Oxoid Ltd., Basingstoke, UK), supplemented with 5 mg/L hemin (Sigma-Aldrich, Saint Louis, MO, USA) and 1 mg/L menadione (Sigma-Aldrich), in an anaerobic chamber (Bactronez-2, Sheldon Manufacturing, Cornelius, OR, USA) at 37 °C and under anaerobic conditions (80% N_2_, 10% CO_2_, and 10% H_2_).

### 4.2. Individuals

Dendritic cells were obtained from 12 healthy non-smoking donors, consecutively enrolled at the Graduate Clinic of the Faculty of Dentistry from Universidad de Chile. The study group consisted of 12 adult individuals (6 males and 6 females) with a mean age of 29.3 ± 4.41 years (ranging from 23 to 39) who did not have periodontal disease as determined by the absence of gingival inflammation, clinical attachment loss, or increased probing pocket depths. Further criteria for individual selection were pregnancy, any type of systemic disease, fever, manifest infections during the last month, symptomatic allergies, abnormal blood cell counts, increased liver enzymes, or intake of any kind of medication, except oral contraceptives and vitamins. An extensive anamnesis was performed, and all data were confirmed with information about medical history and daily habits. The study protocol was approved by the Ethics Committee for Human Research of the North Metropolitan Health Service, Metropolitan Region, Ministry of Health (Protocol code #1/2020) and conducted in compliance with the ethical principles of the World Medical Association Declaration of Helsinki. All subjects provided IRB-approved written consent before blood donation.

### 4.3. Monocyte-Derived Dendritic Cell Generation

From each individual, 80 mL of venous blood was obtained by a brachial puncture to isolate peripheral blood mononuclear cells (PBMCs) using a standard centrifugation protocol (Ficoll-Paque Plus; Amersham Pharmacia Biotech, Uppsala, Sweden). Monocytes were purified from PBMCs by identifying their specific cell surface marker CD14 using a magnetic-cell-sorting procedure (MACS; Miltenyi Biotec, Bergisch Gladbach, Germany). PBMCs were washed twice in PBS and incubated with a microbeads-conjugated monoclonal anti-CD14 antibody for 15 min at 4 °C. After being washed once in PBS, the cells were resuspended, loaded onto large-size (LS) separation columns, and selected by applying a magnetic field. Then, the purified CD14^+^ monocytes were flushed out from the LS columns, counted, and immediately subjected to a standardized protocol of dendritic cell differentiation [64]. Briefly, 10^6^ cells/mL were cultured in 3 mL RPMI-1640 medium (Gibco Invitrogen Corp., Grand Island, NY, USA) containing 10% fetal bovine serum (Gibco Invitrogen Corp.), 20 ng/mL of rhGM-CSF (R&D Systems Inc., Minneapolis, MN, USA), and 20 ng/mL of rhIL-4 (R&D Systems Inc.) for 6 days at 37 °C, refreshing the supplementary cytokines every two days. In each experimental step, cell viability was promptly evaluated by trypan blue exclusion (Luna II, Logos Biosystems, Annandale, VA, USA). For each individual, all experiments were performed separately.

### 4.4. Dendritic Cell Infection

The resulting dendritic cells were infected with the different strains of *P. gingivalis* at a multiplicity of infection MOI = 100 for 2 days, in 6-well culture plates containing 3 mL RPMI-1640 medium supplemented with 10% fetal bovine serum. As a negative control, non-infected dendritic cells were used. Bacterial growth curves were constructed as previously described to carry out cell infections with a known number of bacteria [46]. For this, bacteria were inoculated in 10 mL of liquid brain-heart infusion medium (BD, Le Pont de Claix, France), supplemented with 5 mg/mL of hemin and 1 mg/mL of menadione, until reaching an optical density of 0.05 measured by spectrophotometry at 550 nm (Spectronic 20; Bausch & Lomb, Rochester, NY, USA). For 7 days, optical densities were measured at different culture time points, and bacterial samples were taken to determine the number of colony-forming units (CFUs). The experiment was stopped when the bacteria reached the stationary growth phase. Optical density data versus culture time or CFUs were obtained and plotted. For dendritic cell infections, bacteria were obtained from the exponential growth phase; thus, dendritic cell stimulation was carried out with a reliable number of live bacteria, preserving their full immunogenic potential.

### 4.5. Flow Cytometry Analysis

Phenotyping for monocyte purification, dendritic cell differentiation, and bacteria-induced dendritic cell maturation was performed by flow cytometry using scatter parameters and pan-surface markers. Table 1 shows the details of the monoclonal antibodies used for the sequential identification of the following extracellular markers: CD45 (a pan-leukocyte marker), CD3 (a pan-T-cell marker), CD4 (a helper T-cell marker), CD8 (a cytotoxic T-cell marker), CD14 (a monocyte marker), CD1a (an immature dendritic cell marker), CD209 (a specific monocyte-derived dendritic cell marker), CD83 (a dendritic cell maturation marker), CD80, and CD86 (costimulatory signals necessary for T-lymphocyte activation during antigen presentation). Briefly, cells were stained with 10 µL of each previously diluted antibody for 30 min at 4 °C in the dark. Then, cells were washed twice in PBS and resuspended in 300 mL of fresh PBS to be analyzed. Cell viability was determined using the Zombie UV™ Fixable Viability kit (Biolegend, San Diego, CA, USA). The CD14^+^, CD1a^+^, CD209^+^, CD83^+^, CD80^+^, and CD86^+^ cells were quantified in a flow cytometer (LSR Fortessa X-20, Becton Dickinson Immunocytometry Systems) using a sequential gating strategy according to their forward- and side-scatter (FS/SS) characteristics, live/dead staining, and CD45, CD3, CD4, and CD8 markers. Negative cell populations were determined using isotype-matched control antibodies.

### 4.6. Expression of Cytokines, Transcription Factors, and Toll-like Receptors

Infected dendritic cells were recovered, washed twice in PBS, and counted using an automated cell counter (Luna II, Logos Biosystems). Total cytoplasmic RNA was purified from dendritic cells using an ice-cold lysis buffer containing 0.5% Igepal^®^ CA-630 (Sigma-Aldrich). Then, the first-strand cDNA was synthesized using a reverse-transcription kit following the manufacturer’s instructions (SuperScript III^®^; Invitrogen, Grand Island, NY, USA). From 50 ng cDNA, the mRNA expression levels for the cytokines IL-1β, IL-6, IL-10, IL-12p35, IL-23, IFN-γ, TNF-α, and TGF-β1, the transcription factors IRF4, IRF8, NOTCH2, and BATF3, and the receptors TLR2 and TLR4 were quantified using the appropriate primers (Table 2) and a qPCR reagent (KAPA™ SYBR^®^ Fast qPCR; KAPA Biosystems, Woburn, MA, USA). In a qPCR apparatus (StepOnePlus^®^; Applied Biosystems, Singapore), the following amplification protocol was used: a first step of 95 °C for 3 min, followed by 40 cycles of 95 °C for 3 s and 60 °C for 30 s. To detect non-specific product formation and false-positive amplification, a final melt curve of 95 °C for 15 s, 60 °C for 1 min, and 95 °C for 15 s was performed. The 18S rRNA expression levels were used as an endogenous control for relative quantification.

### 4.7. Secretion of Cytokines

Dendritic cell supernatants were collected, cell detritus was removed by centrifugation for 10 s at 14,000× *g*, and samples were stored at −80 °C until their analysis. The secreted IL-1β, IL-6, IL-23, and TNF-α levels were determined using specific ELISA kits (Quantikine^®^ ELISA Kits; R&D Systems Inc., Minneapolis, MN, USA), following the manufacturer’s recommendations. Briefly, 100 µL of standards and dendritic cell supernatants were added in the respective wells in duplicate, and after following the protocols described in each data sheet, the generated colorimetric changes were measured in an automated plate spectrophotometer (Synergy™ HT, Bio-Tek Instrument Inc., Winooski, VT, USA).

### 4.8. Statistical Analysis

The sample size was determined using the G*Power 3.1 software (Heinrich Heine Universität Düsseldorf, Düsseldorf, Germany), with a 5% significance level (α = 0.05) and an 80% statistical power (1-β = 0.80). Considering IL-23 analyzed by ELISA as a strong variable, the sample size was calculated to detect concentration differences of at least 170 pg/mL between the groups of cells induced with the W50 or GPA strains, with a standard deviation of 195 [46]. Since each blood donor provides cells for each experimental condition, a group of 12 individuals was finally obtained.

Data were processed and statistically analyzed using R software version 4.1.3 (2022). The flow cytometry data were plotted and analyzed using the ggCyto and flowStats packages [65,66]. The qPCR data were calculated using the 2^−ΔΔCt^ method (StepOne v.2.2.2 software; Applied Biosystems, Singapore). The ELISA results were calculated using a logistic equation of 4 parameters. The normality of data distribution was established using the Kolmogorov-Smirnov test. Differences among the different experimental conditions were determined using one-way ANOVA and Bonferroni post hoc tests. Statistical significance was considered when α < 0.05.

## 5. Conclusions

The encapsulated wild-type W50 strain, belonging to serotype K1 of *P. gingivalis*, induced higher production of IRF4 and NOTCH2 and IL-1β, IL-6, IL-23, and TNF-α compared with its isogenic mutants GPA and GPC defective in the extracellular capsule. Therefore, this encapsulated *P. gingivalis* strain showed an increased capacity to trigger Th1 and Th17 response patterns in infected human dendritic cells.

## Figures and Tables

**Table 1:** Monoclonal antibodies and cell viability kit used for the flow cytometry analysis.

Antibody	Clone	Fluorochrome	Dilution	Supplier	Code
anti-CD1a	HI149	APC-Cy7 ^2^	1:400	Biolegend ^7^	300126
anti-CD3	OKT3	eFluor 660	1:800	eBioscience ^8^	50003741
anti-CD4	RPA-T4	BUV395 ^3^	1:200	BD Biosciences ^9^	564724
anti-CD8	SK1	BV510 ^4^	1:200	Biolegend	344732
anti-CD14	M5E2	BV650	1:200	Biolegend	301835
anti-CD45	2D1	Alexa Fluor 700	1:800	eBioscience	56945941
anti-CD80	W17149D	PerCP-Cy5.5 ^5^	1:400	Biolegend	375412
anti-CD83	HB15e	BV711	1:400	Biolegend	305333
anti-CD86	IT2.2	PE-Cy7 ^6^	1:400	eBioscience	25086941
anti-CD209	9E9A8	BV421	1:400	Biolegend	330118
Cell viability kit ^1^	--	BUV496	1:1000	Biolegend	423108

^1^ Cell viability kit: Zombie UV™ Fixable Viability kit; ^2^ APC-Cy7: Allophycocyanin-Cyanine7; ^3^ BUV: Brilliant Ultra Violet™; ^4^ BV: Brilliant Violet™; ^5^ PerCP-Cy5.5: Peridinin-Chlorophyll-protein-Cyanine5.5; ^6^ PE-Cy7: Phycoerythrin-Cyanine7; ^7^ Biolegend, San Diego, CA, USA; ^8^ eBioscience, San Diego, CA, USA; ^9^ BD Biosciences, San Jose, CA, USA.

**Table 2:** Forward and reverse primers used for cytokine, transcription factor, TLR, and 18S rRNA amplifications by qPCR.

mRNA	Forward Primer	Reverse Primer
IL-1β	ctgtcctgcgtgttgaaaga	ttgggtaatttttgggatctaca
IL-6	gcccagctatgaactccttct	gaaggcagcaggcaacac
IL-10	tgggggagaacctgaagac	ccttgctcttgttttcacagg
IL-12p35	cactcccaaaacctgctgag	tctcttcagaagtgcaagggta
IL-23	agcttcatgcctccctactg	ctgctgagtctcccagtggt
IFN-γ	ggcattttgaagaattggaaag	tttggatgctctggtcatctt
TNF-α	cagcctcttctccttcctgat	gccagagggctgattagaga
TGF-β1	cacgtggagctgtaccagaa	cagccggttgctgaggta
IRF4 ^1^	gacaacgccttacccttcg	aggggtggcatcatgtagtt
IRF8 ^2^	tggggatgatcaaaaggagcc	aactggctggtgtcgaagac
NOTCH2 ^3^	cagttacccacccacaggtc	ccatacaggcagtcaatggaa
BATF3 ^4^	agacccagaaggctgacaag	ctccgcagcatggtgttt
TLR2	ctctcggtgtcggaatgtc	aggatcagcaggaacagagc
TLR4	ccctcccctgtaccttct	tccctgccttgaataccttc
18S rRNA	ctcaacacgggaaacctcac	cgctccaccaactaagaacg

^1^ IRF4: interferon regulatory factor 4; ^2^ IRF8: interferon regulatory factor 8; ^3^ NOTCH2: neurogenic locus notch homolog protein 2; ^4^ BATF3: basic leucine zipper ATF-like transcription factor 3.

## Data Availability

The original contributions presented in the study are included in the article, further inquiries can be directed to the corresponding author/s.
